# Association between genetic risk scoring for schizophrenia and bipolar disorder with regional subcortical volumes

**DOI:** 10.1038/tp.2015.195

**Published:** 2015-12-08

**Authors:** X Caseras, K E Tansey, S Foley, D Linden

**Affiliations:** 1 MRC Centre for Neuropsychiatric Genetics and Genomics, Institute of Psychological Medicine and Clinical Neurosciences, Cardiff University, Cardiff, UK; 2 MRC Integrative Epidemiology Unit, School of Social and Community Medicine, Faculty of Medicine & Dentistry, University of Bristol, Bristol, UK; 3 Cardiff University Brain Research Imaging Centre (CUBRIC), School of Psychology, Cardiff University, Cardiff, UK

## Abstract

Previous research has shown coincident abnormal regional brain volume in patients with schizophrenia (SCZ) and bipolar disorder (BD) compared with controls. Whether these abnormalities are genetically driven or explained by secondary effects of the disorder or environmental factors is unknown. We aimed to investigate the association between genetic risk scoring (GRS) for SCZ and BD with volume of brain areas previously shown to be different between these clinical groups and healthy controls. We obtained subcortical brain volume measures and GRS for SCZ and BD from a sample of 274 healthy volunteers (71.4% females, mean age 24.7 (s.d. 6.9)). Volume of the globus pallidus was associated with the shared GRS between SCZ and BD, and also with the independent GRS for each of these disorders. Volume of the amygdala was associated with the non-shared GRS between SCZ and BD, and with the independent GRS for BD. Our results for volume of the globus pallidus support the idea of SCZ and BD sharing a common underlying neurobiological abnormality associated with a common genetic risk for both these disorders. Results for volume of the amygdala, though, would suggest the existence of a distinct mechanism only associated with genetic risk for BD. Finally, the lack of association between genetic risk and volume of most subcortical structures suggests that the volumetric differences reported in patient–control comparisons may not be genetically driven, but a consequence of the disorder or co-occurring environmental factors.

## Introduction

Recent neuroimaging research has shown bipolar disorder (BD) to be associated with brain structural abnormalities. Different meta-analytical efforts have shown presence of enlarged lateral ventricles and globus pallidus and reduced prefrontal volume in BD patients compared with healthy controls.^1, 2, 3^ More recently, the ENIGMA (Enhancing NeuroImaging Genetics through Meta-Analysis) consortium has performed the largest study of subcortical brain volumes in BD to date—over 1500 patients and 2500 controls—replicating the enlargement of lateral ventricles in patients with BD, but not that of the globus pallidus, and also reporting decreased volume of the thalamus, amygdala and hippocampus.^4^

Overlapping results have been reported in structural brain imaging studies of schizophrenia (SCZ). In a meta-analysis of 58 studies, Wright *et al.*^5^ found evidence in SCZ for enlarged lateral ventricles and globus pallidus, and reduced volume of amygdala, hippocampus, thalamus and caudate. The ENIGMA consortium, with a sample of over 2000 cases and controls, also reported enlarged lateral ventricles and globus pallidus, and reduced volumes of amygdala, hippocampus, thalamus and accumbens.^6^

Fewer studies have compared participants diagnosed with BD and SCZ directly, but the results of those point again towards a very important coincidence in subcortical and cortical gray matter abnormalities between both disorders, with SCZ being associated with more extensive abnormalities and greater effect sizes^3, 7, 8^ and therefore suggesting the differences between both these disorders being quantitative rather than qualitative. This correspondence in brain anatomical abnormalities is not surprising considering the important overlap between both disorders in terms of their clinical presentation and associated cognitive deficits.^9, 10^ Moreover, recent genetic research has also highlighted a substantial overlap in common genetic variation associated with both disorders.^11, 12^ These results question the classical division set by Kraepelin more than a century ago between the two types of psychosis, suggesting that both disorders might represent differences in the intensity of phenotype alterations caused by the same underlying neurobiological mechanisms.

However, whether the aforementioned anatomical abnormalities predate the onset of the disorder, are a consequence of it, or perhaps reflect pharmacological effects of the treatment that these patients commonly receive, is a key question that remains unanswered. It is frequently assumed that if predating the disorder, these abnormalities would arise from genetic factors, but it could also be the case that they reflect exposure to environmental factors that contribute to both disorders. To address this issue, some studies have included non-affected relatives of BD and SCZ patients in their design.^13, 14, 15^ Here, the premise is that the unaffected relatives of people with the disorder are at higher genetic risk than the population average but are not subject to secondary effects of the disorder itself (including its treatment). Consequently, if the unaffected relatives have similar abnormalities to cases, it can be concluded that these are more likely to be genetically driven than environmentally or pharmacologically caused. However, to date these studies have mostly failed to replicate the aforementioned coincidence in anatomical abnormalities between SCZ and BD, most likely due to their smaller sample sizes and the consequent limitation in statistical power.

Developments in genetics have recently allowed a new method for investigating the relationship between genetic risk and brain volumes that relies on a quantitative direct measurement of risk rather than categorical stratification by family history. This method, known as genetic risk scoring (GRS), uses risk alleles derived from genome-wide association studies (GWAS) studies to quantify the common risk allele burden carried by individuals for a given disorder based on the average number of independent risk alleles, weighted by their effect size. In the present study, we aimed to investigate the association between the volumes of brain regions previously shown to be abnormal in SCZ and BD (that is, thalamus, amygdala, hippocampus, globus pallidus, caudate and lateral ventricles) and GRS for both these disorders in a large sample of healthy participants with no history of mental disorders and free of any psychotropic medication. Assuming that structural brain abnormalities reported in BD and SCZ are consequences of genetic risk factors that are common to both disorders, we predict the volume of these subcortical areas to be associated with the shared genetic risk for both disorders, and less so with their distinct genetic risk. The fact that the sample only includes participants with no personal history of BD or SCZ precludes any secondary influences of disease course or treatment on brain volume.

## Materials and methods

### Participants

Brain scans used in this study were obtained from a repository of neuroimaging and genetic data obtained between 2009 and 2014 from healthy subjects recruited at Cardiff University Brain Research Imaging Centre (CUBRIC). The original sample consisted of 319 individuals that contributed neuroimaging and genetic data. Participants were recruited if they had no current or past history of psychiatric disorders according to clinical screening, were aged between 18 and 55, and had no history of neurological or medical conditions that could have affected brain anatomy or incurred a scanning risk (for example, head trauma, epilepsy, cardiac conditions).

### Genotyping and quality control

Genomic DNA was obtained from saliva using Oragene OG-500 saliva kits (DNA Genotek, Kanata, ON, Canada). Genotyping was performed using custom Illumina HumanCoreExome-24 BeadChip genotyping arrays that contained probes for 5 70 038 genetic variants (Illumina, San Diego, CA). Quality control was implemented in PLINK.^16^ Individuals were excluded for any of the following reasons: (1) no match between reported and molecular sex; (2) cryptic relatedness of up to third degree relatives as ascertained using identity by descent; (3) genotyping completeness <97% (4) and non-European ancestry. The latter was detected as outliers in iterative EIGENSTRAT analyses of a linkage disequilibrium pruned data set.^17^ Single nucleotide polymorphisms (SNPs) were excluded for any of the following reasons: (1) minor allele frequency <1% (2) genotype call rate <98% and (3) Hardy–Weinberg equilibrium *P*-value<1e−04. Genotypes were imputed using the pre-phasing/imputation stepwise approach implemented in IMPUTE2/SHAPEIT^18, 19^ and 1000 Genomes (December 2013, release 1000 Genomes haplotypes Phase I integrated variant set) as the reference data set.

After quality control, the sample was reduced to 274 participants (195 (71.4%) females, mean age 24.77 (s.d. 6.86)). About 2 96 832 genotyped SNPs passed quality controls and resulted in a data set of 74 13 342 imputed SNPs.

### MRI data acquisition and processing

All magnetic resonance imaging (MRI) data were acquired in a GE Sigma HD × 3T scanner (GE Healthcare, Milwaukee, WI, USA) at CUBRIC. Anatomical images of the brain consisted of 3D fast spoiled gradient echo images obtained at TR=7.9 ms, TE=3.0  ms, inversion time=450 ms, flip angle=20, acquisition matrix=256 (AP) × 192 (LR) × 172 (SI), 1 mm isotropic voxels.

Brain images were automatically segmented using FreeSurfer software (http://surfer.nmr.mgh.harvard.edu) and the resulting outputs quality controlled following a publically available protocol from ENIGMA (http://enigma.ini.usc.edu/). Whenever a region-of-interest (ROI) was detected as inadequately segmented, its metric was declared missing and excluded from the analysis.

### Genetic risk score

GRS methodology follows the procedure described by the International Schizophrenia Consortium.^11^ We calculated GRS using the results from four GWAS as our training data sets: (1) SCZ and BD cases versus controls (GRS-SCZ&BD);^20^ (2) SCZ cases versus BD cases (GRS-SCZvsBD);^20^ (3) SCZ cases versus controls (GRS-SCZ);^21, 22^ and (4) BD cases versus controls (GRS-BD).^22, 23^ For the GRS-SCZ and GRS-BD, we used results generated by the Cross-Disorder Group of the Psychiatric Genomics Consortium (PGC),^22^ which removed any overlapping controls from the data sets, ensuring the data sets were completely independent. Despite the existence of recent larger GWAS data for SCZ, for consistency we calculated the GRS-SCZ from the initial iterations of the PGC data for SCZ.^21^ The main focus of our analysis are the composite scores GRS-SCZ&BD and GRS-SCZvsBD and those are only available from the initial iterations of the PGC data; likewise, currently GRS-BD can only be obtained from the initial iteration of the PGC data.

For all analyses, SNPs were removed if they had a low minor allele frequency (MAF<0.01), and were subsequently pruned for linkage disequilibrium using the (*r*^2^<0.25) clumping function (—clump) in PLINK^16^ removing SNPs within 500 kb (—clump-kb) and *r*^2^>0.25 (—clump-*r*^2^) with a more significantly associated SNP. We used the score command in PLINK to calculated GRS,^16^ which were calculated by summing the number of susceptibility alleles of each index SNP weighted by the logarithm of the SNP odds ratios. Six *P*-value thresholds (*P*_T_<1 × 10^−5^, 1 × 10^−4^, 0.01, 0.1, 0.3, 0.5) for SNP association were used.

### Association analyses

All subcortical volumes (averaged left and right hemispheres) and GRS considered here followed the normal distribution. GRS were entered as regressors of interest in linear regression analyses—after covariates have been entered into the model—with gray matter volume of each ROI as dependent variable. Age at scan only correlated with the volume of the lateral ventricles and was therefore only included as covariate in the analyses for this ROI. Gender and intracranial volume showed significant association with volume of all the ROIs considered here and were included as covariates in all subsequent analyses. According to our hypotheses, we investigated first the association of GRS-SCZ&BD and GRS-SCZvsBD with volume of the preselected ROIs. Where significant effects were found, we investigated the association with the disorder-specific GRS (that is GRS-SCZ and GRS-BD) to ascertain that the effect was not driven by the sole association with one of the phenotypes. Based on Sham and Purcell's^24^recommendations for multiple testing correction in dependent tests, we applied a *post hoc* correction for multiple comparisons by performing 10 000 permutations of each linear regression analysis resulting in a nominal *P<*0.05. These were performed using R.^25^ The permuted *P*-value was calculated as the total number of times a permutation resulted in a *P*-value less than the observed *P*-value, divided by the number of permutations. If the permuted *P*-value was *P<*0.05, then we considered the association significant after correction for multiple testing.

## Results

**Figure 1:**
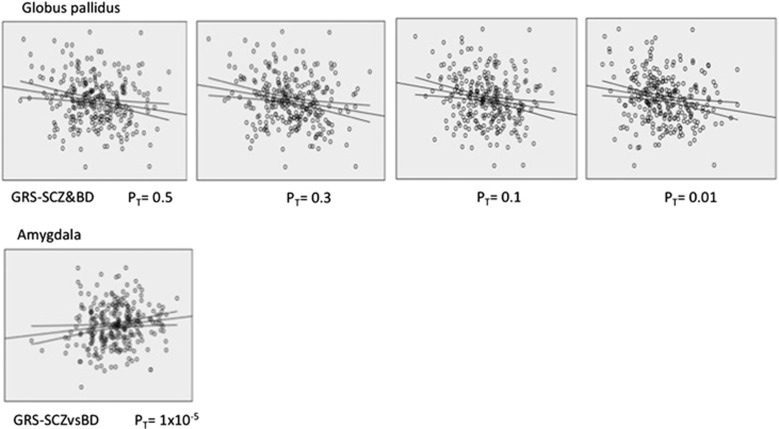
Scatterplots representing the significant association between volume of the globus pallidus and the shared genetic risk scoring for schizophrenia and bipolar disorder calculated at different *P*_T_ levels (top row), and between volume of the amygdala and the non-shared genetic risk scoring for schizophrenia and bipolar disorder obtained at *P*_T_ 1 × 10^−5^ (bottom row). BD, bipolar disorder; GRS, genetic risk score; SCZ, schizophrenia.

The GRS-SCZ&BD calculated at *P*_T_<0.01, 0.1, 0.3 and 0.5 was negatively associated with volume of the globus pallidus. GRS-SCZ&BD at *P*_T_<1 × 10^−4^ was negatively correlated with volume of the thalamus, although this did not survive correction for multiple comparison (Table 1 and Figure 1). To ascertain whether this effect in the globus pallidus was driven mainly by only one of the phenotypes, the association between volume of this ROI and the GRS-SCZ and GRS-BD was calculated. As shown in Table 2, the volume of the globus pallidus showed significant association with both genetic scores at different *P*_T_ levels.

The GRS-SCZvsBD only showed significant association—negative correlation—when calculated at *P*_T_<1 × 10^−5^ with volume of the amygdala (Table 1 and Figure 1). Analyses with the disease-specific scores revealed that volume of the amygdala was negatively associated with GRS-BD but not with GRS-SCZ (Table 2).

## Discussion

This study aimed to investigate the relationship between composite measures of genetic risk for SCZ and BD with the volume of subcortical brain structures that have previously been shown to differ between cases and controls in clinical samples of SCZ and BD, in a sample of healthy participants. Our results showed the volume of the globus pallidus to be associated with the genetic risk for both these disorders. Interestingly, the predictive value—change in variance explained (Δ*R*^2^)—of the combined SCZ&BD GWAS on the volume of this structure was larger than the predictive value of the risk for each disorder considered separately. This suggests that the volume of the globus pallidus is associated with genetic variants that influence risk for both SCZ and BD, rather than with non-shared genetic variants. Contrary, the volume of the amygdala was only associated with genetic risk for BD; although in this case, fewer associations with GRS calculated at different *P*_T_ values were found, making this finding less robust.

Previous research has shown the globus pallidus to be larger in BD and SCZ patients compared with healthy controls.^3, 4, 6^ Here, we show volume of this brain structure is negatively associated with genetic risk for these disorders in a sample of healthy participants. Given that our participants were un-medicated and did not suffer from any psychiatric condition, this association could not be mediated by secondary effects of these disorders, or by the effect of psychotropic medication. Moreover, we show that the volume of this brain structure is associated with that fraction of genetic risk that is shared across BD and SCZ. Interestingly, we found an inverse relationship between genetic risk for these disorders and the volume of the globus pallidus; that is, the higher the risk added by common genetic variants the smaller the volume of this structure. Antipsychotic medication has shown to increase the volume of basal ganglia structures.^26^ The ENIGMA consortium results for BD^4^ show enlarged volume of the globus pallidus in patients taking typical antipsychotics compared with healthy controls and also compared with patients under other class of drugs/no-drugs. Similarly, those taking atypical antipsychotics showed enlarged volume than healthy controls and patients under other class of drugs/no-drugs, albeit in this case not reaching statistical significance.^4^ The ENIGMA SCZ group also showed that volume of the globus pallidus was positively associated with illness duration.^6^ Summarizing, the present results show volume of the globus pallidus to be associated with the shared genetic risk for BD and SCZ; the fact that we find an inverse relationship while clinical studies show enlargement in clinical samples relative to controls could be explained by the influence of factors like illness duration or antipsychotic medication. This stresses the need to cautiously interpret the results from any comparisons between clinical and control samples, or studies of joined clinical and healthy samples, where many factors associated with the pathology might conceal the results.

Our results also suggest amygdala volume to be negatively associated with genetic risk for BD, but not for SCZ. Although in this case the association follows the expected direction—that is, decreasing volume as genetic risk increases—the lack of association with genetic risk for SCZ or with the added risk for both disorders was unexpected. Previous research has consistently shown reduced amygdala volume in SCZ patients compared with controls, with these studies presenting larger effect sizes than equivalent studies in BD.^5, 6^ Unlike in the analyses with globus pallidus, though, the significant association between GRS-SCZvsBD and amygdala volume was limited to one GRS value (that is, *P*_T_ 1 × 10^−5^), and to only two GRS values when using the GRS-BD scores (that is, *P*_T_ 1 × 10^−5^ and *P*_T_ 0.01). The limited number of significant associations in this case could suggest the possibility of these representing a type I error, and further replication would be required before firm conclusions can be drawn.

The lack of association between most subcortical volumes considered here and GRS for SCZ and BD suggests that the abnormalities observed in these structures in clinical studies comparing patients and controls are not a consequence of disorder liability. Whether they reflect environmental exposures on the causal pathway to disease, or whether they are consequences of the disorder cannot be answered by the present study. The fact that some of these abnormalities have been shown in non-affected relatives of SCZ or BD patients, led to the conclusion that these were associated with genetic risk. However, these might well still be explained by shared environmental factors between patients and their close relatives. For example, Boos *et al.*^27^ showed in a meta-analysis that like patients, non-affected SCZ relatives showed reduced hippocampal volume compared with healthy controls. However, it has been recognized that the family environment of SCZ patients presents with high levels of stress,^28^ and that exposure to stress may contribute to reductions in hippocampal volume via hypercortisolemia.^29^ Therefore, reduced hippocampal volume in SCZ patients and their relatives compared with healthy controls with no family history of SCZ could be explained by differences in the levels of stress to which these groups are exposed throughout their lives, rather than to genetic factors.

The report presented here has some strengths and weaknesses that should be noted. Our sample is large for a study homogeneous in terms of recruitment methods, exclusion criteria, magnet, MRI acquisition protocol, data pre-processing, analysis and quality control. The extent to which this affords a relative advantage over larger, but much more heterogeneous, meta-analyses such as those by ENIGMA is, however, not clear. Future researches with similarly homogeneous but larger samples are necessary to validate the present findings. For example, considering the average correlation found here between volume of lateral ventricles and the GRS-SCZ&BD (*r*=0.1), and to maintain a nominal *P*-value of 0.05 and a power of 0.85, a sample size of *n=*717 would be required for that to be significant (StatsToDo; www.statstodo.com/index.php). This is even more relevant with regard to our conclusion that most previously reported volume abnormalities are not a consequence of disorder liability, since lack of association in our study could simply be a consequence of limited statistical power. Also, as previously stated, due to the lack of an equivalent GWAS for BD, we based our GRS calculations on the initial iterations of the PGC data for SCZ.^21^ Since GRS estimated from larger GWAS are meant to be more accurate, our results will require replication once the latest GWAS for BD results are available. Also, these new GRS would be expected to present lower variability within healthy control samples, and again are likely to require larger sample sizes to be powered to reveal any significant association.

In summary, our study reports an association between volume of the globus pallidus and genetic risk for SCZ and BD, and suggests this is driven by alleles that confer risk for both disorders. This result adds further to the possibility that SCZ and BD share a common genetically driven etiological mechanism in line with the dimensional focus of the Research Domain Criteria.^30^ However, we also found a potential distinct association between genetic risk for these disorders and amygdala volume, which will also support the existence of distinct genetically driven etiological mechanism between SCZ and BD. The latter result, though, was less robust and requires more cautious consideration. Importantly, our study also questions whether other repeatedly reported volumetric abnormalities (for example, enlarged lateral ventricles, reduced thalamus) are associated with genetic risk for SCZ and/or BD, rather than being a consequence of the disease or of the exposure to environmental factors.

## Figures and Tables

**Table 1:** Results from association between risk profile score (RPS) for schizophrenia and bipolar disorder cases versus controls (SCZ&BD), schizophrenia cases versus bipolar disorder cases (SCZvsBD) and total volume in *a priori* selected regions-of-interest

*RPS* P_*T*_	*Thalamus* β (P*) Δ*R^*2*^	*Hippocampus* β (P*) Δ*R^*2*^	*Amygdala* β (P*) Δ*R^*2*^	*Globus pallidus* β (P*) Δ*R^*2*^	*Caudate* β (P*) Δ*R^*2*^	*Lateral ventricles* β (P*) Δ*R^*2*^
*SCZ&BD*						
1 × 10^−5^	−0.074 (0.064) 0.005	0.034 (0.477) 0.001	−0.032 (0.515) 0.001	0.023 (0.650) 0.001	−0.043 (0.431) 0.002	0.010 (0.868) <0.001
1 × 10^−4^	**−0.091 (0.021) 0.008**	0.000 (0.993) <0.001	−0.056 (0.250) 0.003	−0.091 (0.069) 0.008	−0.046 (0.396) 0.002	0.036 (0.530) 0.001
0.01	−0.025 (0.534) 0.001	−0.002 (0.971) <0.001	−0.069 (0.161) 0.005	−0.149 (0.003)* 0.022	−0.031 (0.570) 0.001	0.040 (0.486) 0.002
0.1	0.017 (0.660) <0.001	0.019 (0.695) <0.001	−0.018 (0.714) <0.001	−0.151 (0.002)* 0.023	0.005 (0.922) <0.001	−0.076 (0.183) 0.006
0.3	0.020 (0.622) <0.001	−0.027 (0.580) 0.001	−0.023 (0.635) 0.001	−0.162 (0.001)* 0.026	0.016 (0.771) <0.001	−0.059 (0.307) 0.003
0.5	0.024 (0.551) 0.001	−0.036 (0.457) 0.001	−0.021 (0.673) <0.001	−0.144 (0.004)* 0.020	−0.005 (0.925)<0.001	−0.077 (0.178) 0.006
						
*SCZvsBD*						
1 × 10^−5^	−0.007 (0.854) <0.001	0.037 (0.434) 0.001	0.107 (0.029)* 0.011	0.069 (0.166) 0.005	0.058 (0.281) 0.003	0.005 (0.930) <0.001
1 × 10^−4^	−0.050 (0.202) 0.003	0.088 (0.064) 0.008	0.046 (0.348) 0.002	0.000 (0.997) <0.001	0.036 (0.512) 0.001	0.007 (0.909) <0.001
0.01	0.057 (0.146) 0.003	−0.005 (0.916) <0.001	0.013 (0.797) <0.001	0.002 (0.976) <0.001	−0.052 (0.335) 0.003	−0.006 (0.917) <0.001
0.1	0.070 (0.078) 0.005	0.030 (0.536) 0.001	−0.042 (0.394) 0.002	−0.031 (0.534) 0.001	0.053 (0.333) 0.003	−0.028 (0.629) 0.001
0.3	0.046 (0.248) 0.002	−0.009 (0.849) <0.001	−0.049 (0.318) 0.002	−0.015 (0.765) <0.001	0.046 (0.398) 0.002	−0.022 (0.696) <0.001
0.5	0.049 (0.217) 0.002	−0.011 (0.819) <0.001	−0.039 (0.431) 0.001	−0.028 (0.575) 0.001	0.049 (0.372) 0.002	−0.019 (0.742) <0.001

*β*, beta value for the RPS in the regression equation after covariates have been included; *P*, significance level associated to the *β* value, all *P*-values reported are nominal; Δ*R*^2^, change in variance explained added by the RPS.

Bold results signify those with nominal *P*-values<0.05. Asterisk indicates those with permuted *P*-values<0.05.

**Table 2:** Results from association between risk profile score (RPS) for schizophrenia (SCZ) cases versus controls and bipolar disorder (BD) cases versus controls with total volume in amygdala and globus pallidus

*RPS* P_*T*_	*Amygdala* β (P*) Δ*R^*2*^	*Globus pallidus* β (P*) Δ*R^*2*^
*SCZ*		
1 × 10^−5^	−0.037 (0.446) 0.001	0.039 (0.434) 0.002
1 × 10^−4^	−0.010 (0.837) <0.001	−0.004 (0.943) <0.001
0.01	−0.068 (0.168) 0.005	**−0.142 (0.004) 0.020**
0.1	−0.018 (0.714) <0.001	−0.095 (0.056) 0.009
0.3	0.004 (0.937) <0.001	−0.104 (0.039) 0.011
0.5	0.002 (0.974) <0.001	**−0.115 (0.022) 0.013**
		
*BD*		
1 × 10^−5^	**−0.142 (0.004) 0.020**	−0.050 (0.314) 0.003
1 × 10^−4^	−0.086 (0.079) 0.007	−0.020 (0.694) <0.001
0.01	**−0.098 (0.045) 0.010**	**−0.125 (0.012) 0.016**
0.1	0.017 (0.724) <0.001	**−0.102 (0.042) 0.010**
0.3	−0.016 (0.740) <0.001	−0.066 (0.188) 0.004
0.5	−0.043 (0.376) 0.002	−0.066 (0.185) 0.004

*β*, beta value for the RPS in the regression equation after covariates have been included; *P*, significance level associated to the *β* value, all *P*-values reported are nominal; Δ*R*^2^, change in variance explained added by the RPS.

Bold results signify those with nominal *P*-value<0.05.
